# Multiple-Replicon Resistance Plasmids of *Klebsiella* Mediate Extensive Dissemination of Antimicrobial Genes

**DOI:** 10.3389/fmicb.2021.754931

**Published:** 2021-10-27

**Authors:** Xue Wang, Jianan Zhao, Fang Ji, Han Chang, Jiao Qin, Chenglin Zhang, Guocheng Hu, Jiayue Zhu, Jianchun Yang, Zhongxin Jia, Gang Li, Jianhua Qin, Bin Wu, Chengmin Wang

**Affiliations:** ^1^Guangdong Key Laboratory of Animal Conservation and Resource Utilization, Guangdong Public Laboratory of Wild Animal Conservation and Utilization, Institute of Zoology, Guangdong Academy of Science, Guangzhou, China; ^2^College of Veterinary Medicine, Agricultural University of Hebei, Baoding, China; ^3^Institute of Zoology, Chinese Academy of Sciences, Beijing, China; ^4^Beijing Key Laboratory of Captive Wildlife Technologies, Beijing Zoo, Beijing, China; ^5^South China Institute of Environmental Sciences, Ministry of Ecology and Environment, Guangzhou, China; ^6^School of Bioengineering, East China University of Science and Technology, Shanghai, China

**Keywords:** multiple-replicon plasmids, *Klebsiella*, antibiotic resistance genes, comparative genomic analysis, extensive dissemination

## Abstract

Multiple-replicon resistance plasmids have become important carriers of resistance genes in Gram-negative bacteria, and the evolution of multiple-replicon plasmids is still not clear. Here, 56 isolates of *Klebsiella* isolated from different wild animals and environments between 2018 and 2020 were identified by phenotyping *via* the micro-broth dilution method and were sequenced and analyzed for bacterial genome-wide association study. Our results revealed that the isolates from non-human sources showed more extensive drug resistance and especially strong resistance to ampicillin (up to 80.36%). The isolates from *Malayan pangolin* were particularly highly resistant to cephalosporins, chloramphenicol, levofloxacin, and sulfamethoxazole. Genomic analysis showed that the resistance plasmids in these isolates carried many antibiotic resistance genes. Further analysis of 69 plasmids demonstrated that 28 plasmids were multiple-replicon plasmids, mainly carrying beta-lactamase genes such as *bla*_CTX–M–__15_, *bla*_CTX–M–__14_, *bla*_CTX–M–__55_, *bla*_OXA–__1_, and *bla*_TEM–__1_. The analysis of plasmids carried by different isolates showed that *Klebsiella pneumoniae* might be an important multiple-replicon plasmid host. Plasmid skeleton and structure analyses showed that a multiple-replicon plasmid was formed by the fusion of two or more single plasmids, conferring strong adaptability to the antibiotic environment and continuously increasing the ability of drug-resistant isolates to spread around the world. In conclusion, multiple-replicon plasmids are better able to carry resistance genes than non-multiple-replicon plasmids, which may be an important mechanism underlying bacterial responses to environments with high-antibiotic pressure. This phenomenon will be highly significant for exploring bacterial resistance gene transmission and diffusion mechanisms in the future.

## Introduction

The global diffusion of resistance genes is usually related to horizontal gene transfer mediated by plasmids. Plasmid conjugation can occur in various species. Conjugated plasmids are important vectors for the transmission of antibiotic resistance gene (ARG) clusters in Gram-negative bacteria (Moran et al., 2019). There are many types of plasmids, which can be divided into several incompatible groups according to the differences in common gene elements involved in replication regulation or division (Novick, 1987), and each type includes multiple incompatible subgroups (Villa et al., 2010). Among the highly diverse carried by *Enterobacteriaceae*, the most common types of replicons include the members of the incompatible groups IncF, IncA/C, IncL/M, IncI1, IncHI2, and IncN (Carattoli, 2009). Incompatible groups such as IncF, IncI, IncA/C, IncL (IncL/M), IncN, and IncH are often considered to be plasmid types that carry a greater number of resistance gene types (Rozwandowicz et al., 2018). IncF is a narrow-host-spectrum plasmid that is widely found in *Enterobacteriaceae*. It can carry a variety of ARGs and plays a major role in the diffusion of specific resistance genes (Carattoli, 2011). In recent years, many studies have shown that the IncF subtype IncFIB/IncFII plasmid is closely related to ARGs (Deng et al., 2011; Wang et al., 2017; Bai et al., 2019; Pérez-Vázquez et al., 2019; Wu et al., 2019; Bougnom et al., 2020). Many resistance genes, such as those encoding cephalosporinase (*bla*_CTX–M_, etc.), carbapenemase (KPC, etc.), aminoglycoside acetylase (aac6′-1B) (Hawkey and Jones, 2009), and the myxomycetin resistance gene MCR-1 (Liu et al., 2016), are mainly carried by plasmids. These resistance plasmids spread in the environment, causing serious public health and safety risks.

In recent years, some multiple-replicon plasmids with complex structures have received increasing attention. The coexistence of multiple-replicon plasmids is more common among IncF-group plasmids (Villa et al., 2010). An analysis of the plasmids p721005 KPC, p504051 KPC, and pA3295 KPC showed that the structure of p721005-KPC/p504051-KPC was composed of a skeleton of the IncR type, a conjugated transfer region, a maintenance region, and a skeleton of the IncFII type. The pA3295 KPC skeleton was formed by the hybridization of an IncFII type skeleton region, a maintenance region with an IncN1 type maintenance region, and a conjugated transfer region (Qu et al., 2019). The fusion of the phage-like IncN1-F33: A-: B-plasmids carrying MCR-1 mediated by the IS26 insertion sequence plays an important role in the process of plasmid recombination (He et al., 2019). Another study found that CG258, which carries the composite plasmid pKPC-1k30/pHN7A8 of the IncFII family, exhibits clonal transmission among many hospitals in China (Shi et al., 2018). Pesesky et al. (2019) believed that transposons might play a major role in the formation of plasmid chimeras, which are abundant and unevenly distributed in the genome or plasmid DNA of prokaryotes. These plasmid chimeras are effective vectors for a variety of ARGs. Therefore, in-depth studies of the relationships between the types of plasmid replicons carried by bacteria and ARGs in ecological environments are of great significance for revealing the response strategies of bacterial populations in antibiotic environments, monitoring epidemiological dynamics, and establishing intervention programs for specific plasmid transmission.

In this study, we obtained six multidrug-resistant *Klebsiella pneumoniae* strains from *Malayan pangolin*. These strains were highly resistant to β-*lactams*, *fluoroquinolones*, and *chloramphenicols* and carried multiple-replicon complex plasmids. To evaluate the prevalence and spread of multiple-replicon plasmids and their association with ARGs, *Klebsiella* sp. isolates obtained from various wild animals, and clinical samples from hospitals were further analyzed. The bacterial genome-wide association study technology was used for data mining to explore further the significance of multiple-replicon plasmids in the survival strategy of bacteria responding to antibiotic environments.

## Materials and Methods

### Bacterial Isolates and Antimicrobial Susceptibility Testing

*Klebsiella* sp. isolates were isolated and collected from 2018 to 2020 (Wang et al., 2020) and preserved in the laboratory, from which 56 *Klebsiella* sp. isolates were selected, including 21 isolates from fresh fecal samples of animals and 35 isolates from human clinical samples (Supplementary Table 1). The pure isolates were revived in Mueller–Hinton broth. The *Klebsiella* sp. isolates were subjected to antimicrobial susceptibility testing using a broth microdilution kit (BIO-KONT, China) to determine the minimum inhibitory concentrations of ampicillin (AMP, 64–2 μg/ml), cefuroxime (CXM, 64–2 μg/ml), cefazolin (CZO, 16–0.5 μg/ml), ceftriaxone (CRO,8–0.25 μg/ml), cefepime (FEP, 64–2 μg/ml), ampicillin/sulbactam (SAM, 64/32–2/1 μg/ml), piperacillin/tazobactam (TZP, 256/4–8/4 μg/ml), meropenem (MEM, 8–0.25 μg/ml), gentamicin (GEN, 32–1 μg/ml), amikacin (AMK, 128–8 μg/ml), chloramphenicol (CHL, 64–2 μg/ml), levofloxacin (LVX, 16–0.5 μg/ml), cotrimoxazole (SXT, 8/152–0.25/4.75 μg/ml), and tigecycline (TGC, 16–0.5 μg/ml); the broth microdilution method was conducted according to guidelines of the Clinical and Laboratory Standards Institute (CLSI). The susceptibility results were interpreted according to the CLSI 2019 guidelines (Clinical and Laboratory Standards Institute [CLSI], 2019), whereas the breakpoint for polymyxin E and TGC followed the European Committee on Antimicrobial Susceptibility Testing V6.0 (The European Committee on Antimicrobial Susceptibility Testing, 2020). *Escherichia coli* ATCC25922 was used for quality control.

### Whole-Genome Sequencing and Analysis

Genomic DNA extracted from 56 *Klebsiella* sp. isolates was subjected to whole-genome sequencing using the Oxford Nanopore Technologies MinION platforms (Biomarker Technologies Co., Ltd., Beijing, China; Ashton et al., 2015; Loman et al., 2015). Sequencing reads, including short-read and long-read data, were assembled using Unicycler 0.4.4 *via* the hybrid strategy (Wick et al., 2017; Li et al., 2018). The plasmid sequences were initially annotated using the RAST server^1^ and corrected manually. The plasmid replicon genotype was identified using PlasmidFinder.^2^ IS elements were identified using ISfinder^3^; comparative analysis was performed, and plasmid maps were generated using Easyfig and BRIG (Alikhan et al., 2011; Sullivan et al., 2011). The phage sequences were screened using PHASTER (Arndt et al., 2016).

### Multiple Locus Sequence Typing

The Multiple Locus Sequence Typing (MLST) assay was performed as previously described (Clinical and Laboratory Standards Institute [CLSI], 2012). Briefly, seven *K. pneumoniae* housekeeping genes (*infB*, *tonB*, *pgi*, *gapA*, *phoE*, *rpoB*, and *mdh*) were extracted from genomes. Alleles and STs were analyzed using the *K. pneumoniae* MLST database.^4^

### Genome Structure Analysis of Multiple-Replicon Plasmids

The similarity between the sequenced plasmids and known plasmids was compared using PlasmidFinder (see text footnote 4) (Carattoli et al., 2014; Clausen et al., 2018): The parameter is Max.*p*-value = 0, Max.distance = 0.04, and Per.Ident ≥ 60%. The incompatible population data sets of similar plasmids were confirmed, and the reporting year and global frequency of similar plasmids were determined according to the PLSDB database. Using the proprietary database of ARGs (card)^5^, the sequencing plasmids were annotated, and the movable elements and other characteristics were annotated according to the results of genome component analysis and functional annotation (NR, GO, UniProt, COG, SwissProt, Pfam, and KEGG public databases, and ISfinder, integral, and Tn number Registry proprietary database). The SNAPGENE software was used to draw the plasmid maps of some plasmids, and pM1026-3Ar.1 was selected as the representative plasmid whose data were used to construct the plasmid fusion pattern map.

### Nucleotide Sequence Accession Numbers

The complete sequences of the chromosome and four plasmids have been submitted to GenBank under accession numbers (Supplementary Table 1).

## Results

### *Klebsiella* sp. Isolates Showed Strong Multidrug Resistance

**FIGURE 1:**
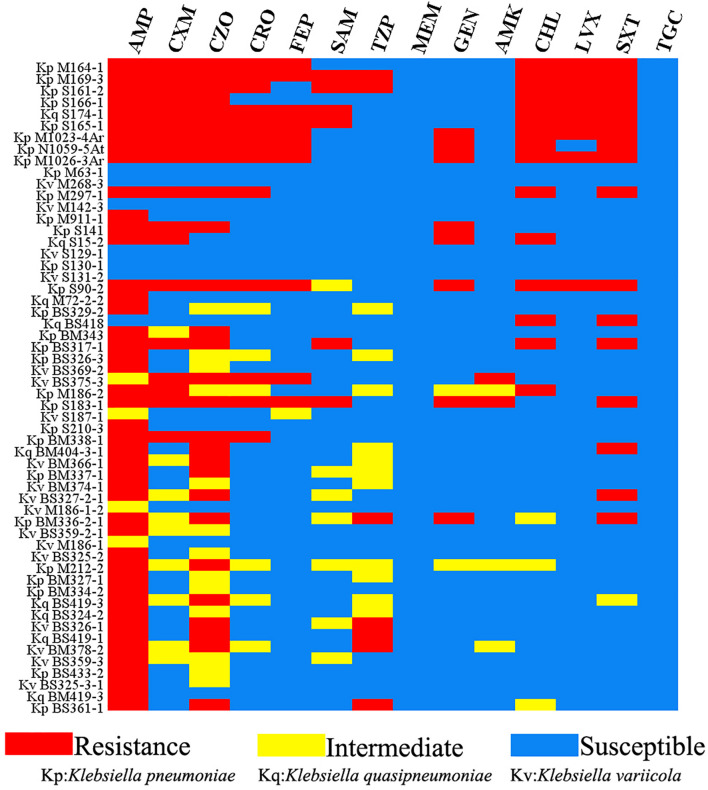
Antimicrobial resistance phenotype of *Klebsiella* isolates in this study. Red: resistance; yellow: intermediate; blue: susceptible. AMP, ampicillin; CXM, cefuroxime; CZO, cefazolin; CRO, ceftriaxone; FEP, cefepime; SAM, ampicillin/sulbactam; TZP, piperacillin/tazobactam; MEM, meropenem; GEN, gentamicin; AMK, amikacin; CHL, chloramphenicol; LVX, levofloxacin; SXT, trimethoprim/sulfamethoxazole; TGC, tigecycline.

The results of antimicrobial susceptibility testing in this study showed that 80.36% (45/56) of *Klebsiella* sp. isolates were resistant to ampicillin. Most isolates (45/56) were multiple drug-resistant (MDR) (resistant to three or more antimicrobial classes; Magiorakos et al., 2012). The isolates from the *M. pangolin* have a high drug resistance rate for cephalosporins, chloramphenicol, levofloxacin, and cotrimoxazole. Only six isolates, M1023-4Ar, N1059-5At, M1026-3Ar, M297-1, S141, and S90-2, from other sources (*yak*, *cow*, *white-lipped deer*, *red kangaroo*, *scarlet breasted parrot*, and *grouse*) were highly resistant to the five drugs mentioned earlier. Among the isolates from human sources, only BS375-3 and S183-1 showed strong resistance to cefuroxime sodium, cefazolin, ceftriaxone, and cefepime (Figure 1). It should be noted that all *Klebsiella* sp. strains showed strong resistance to beta-lactams but were sensitive to MEM and TGC. The phenomenon of the inconsistency between bacterial drug resistance phenotype and genotype does exist, mainly because the mechanism of bacterial drug resistance is extremely complex. For example, M164-1 from *M. pangolin* isolate in this study does not carry the resistance gene of sulfonamides but shows resistance to sulfamethoxazole (Supplementary Table 2). Bacterial drug resistance is a very complex process, and the drug resistance pump system may also play an important role (Supplementary Table 1).

### Analysis of the Correlation Between Multiple-Replicon Plasmids and the Genome of *Klebsiella* sp.

According to the results of 16S rRNA and rMLST identification, of the 56 *Klebsiella* sp. isolates, 29 isolates were identified as *K. pneumoniae*, among which 27 isolates (27/29, 93.10%) carried plasmids; 9 isolates were identified as *Klebsiella quasipneumoniae*, among which 8 isolates (8/9, 88.89%) carried plasmids, and 18 isolates were identified as *Klebsiella variicola* with 5 of these isolates (5/18, 27.78%) carrying plasmids. MLST analysis typing showed there were 33 different ST types, among which 16 ST type isolates carried multiple replicon plasmids, including ST1565 (four isolates), ST1 and ST23 (two isolates each), ST36, ST86, ST101, ST111, ST133, ST231, ST290, ST791, ST1035, ST1662, ST1910, ST2558, and ST3972 (one isolate each) (Supplementary Table 1). In general, the chromosomal DNA of the *Klebsiella* sp. isolates in this study usually carried at least two of the β-lactam resistance genes *bla*_DHA_, *bla*_SHV_, *bla*_LEN_, and *bla*_OKP–B_. In addition, some drug resistance efflux pump genes, including efflux pump genes belonging to the ABC family, MFS family, SMR family, or MATE family, were also carried on chromosomal DNA. Thus, there was no significant correlation between the multiple-replicon plasmids and ST type.

**FIGURE 2:**
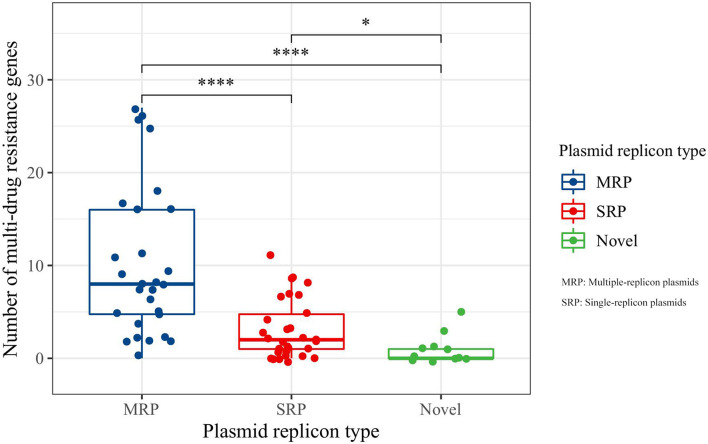
Comparative analysis of antimicrobial resistance genes carried by plasmids. MRP indicates multiple-replicon plasmid; SRP indicates single-replicon plasmid. *Indicates significant difference, ****indicates extremely significant difference.

In the PlasmidFinder database, incompatible group (Inc.) typing is based on the replicon (REP) sequence in plasmid DNA (Carattoli et al., 2014). In this study, 30 of the 69 identified plasmids were single-replicon plasmids, 28 were multiple-replicon plasmids, and 11 were unknown or novel replicon-type plasmids (Supplementary Table 1). Further analysis of the relationship between replicon types and resistance genes showed that the number of resistance genes carried by multiple-replicon plasmids was significantly greater than that carried by non-multiple-replicon plasmids (Figure 2). The proportion of single-replicon plasmids carrying more than five resistance genes was only 26.67%, that of the novel or unknown plasmids was only 9.09%, and that of multiple-replicon plasmids was as high as 75%. The proportion of multiple-replicon plasmids carrying more than 10 resistance genes (*n* ≥ 10) was 35.71%, and a few plasmids carried more than 25 resistance genes. Multiple replicon plasmids mainly carried β-lactamase genes such as *bla*_CTX–M–__15_, *bla*_CTX–M–__14_, *bla*_CTX–M–__55_, *bla*_OXA–__1_, and *bla*_TEM–__1_ and aminoglycosidase genes such as *aac (6*′*)–ib cr*, *aph (6)–id*, and *aph (3″)–ib*. Some plasmids also carried the sulfonamide resistance genes *sul1*, *sul2*, and *sul3*, the tetracycline resistance genes *tetA* and *tetG*, members of the fluoroquinolone resistance *qnr* gene family, and the *floR* gene for chloramphenicol resistance (Supplementary Table 1). Thus, the ability of *Klebsiella* sp. isolates to successfully obtain and carry multiple-replicon plasmids may be an important survival strategy for coping with the environmental pressure of antibiotics.

**FIGURE 3:**
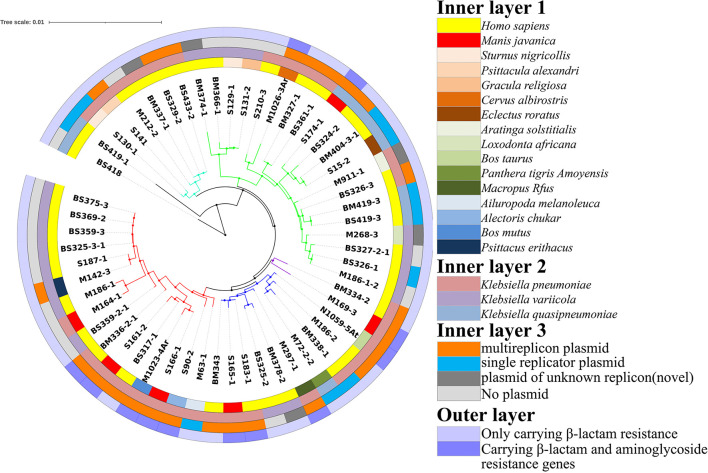
Construction of a phylogenetic tree based on 16S rRNA sequences. Inner layer 1: isolation source; inner layer 2: species; inner layer 3: isolates carrying plasmids; outer layer: plasmids carrying β-lactam resistance genes and aminoglycoside resistance genes.

To evaluate the relationship between *Klebsiella* sp. and multiple-replicon plasmids, the plasmids and drug resistance genes carried by *K. pneumoniae*, *K. variicola*, and *K. quasipneumoniae* were compared and analyzed. Overall, it was found that multiple-replicon plasmids were widely distributed and were mostly carried by *K. pneumoniae* and were rarely carried by *K. quasipneumoniae* and *K. variicola*. The phylogenetic tree of *K. pneumoniae* constructed using 16S rRNA showed that the *K. pneumoniae* isolates were divided into five branches. The MDR *K. pneumoniae* isolates carrying multiple-replicon plasmids were on the same evolutionary branch, suggesting that these isolates showed close homology (Figure 3). It is worth noting that most of the isolates carrying multiple-replicon plasmids came from wild animals rather than from humans (Figure 3), suggesting that wild animals may be important reservoir hosts for MDR bacteria. Therefore, *K. pneumoniae* is considered to be an important host of multiple-replicon plasmids, and wild animals are also important hosts of multidrug-resistant *K. pneumoniae*.

### Gene Composition and Skeleton Analysis of Multiple-Replicon Plasmids

In this study, 28 multiple-replicon plasmids were further analyzed, including eight IncFIB/IncFII/(IncQ1), five IncFIB/IncHI1B/(IncQ1), four IncFII/IncR, four IncHI1B/RepB, two IncFII/IncQ1, one IncFIA/IncFIB/IncFII/IncFII/IncFII, one IncFIA/IncFII, one IncFIA/IncR, one IncFII/IncHI1B/IncR/IncR/RepB, and one IncN/IncN plasmid (Supplementary Table 1). With the exception of the IncFII/IncR and IncFIA/IncFII plasmids, all other multiple-replicon plasmids listed earlier were found to carry many resistance genes. When the number of resistance genes on plasmids increased, the base number of plasmids also increased significantly, but the total G + C content of the plasmids did not increase significantly. As mentioned previously, these plasmids mainly come from natural hosts consisting of a variety of ST-type *K. pneumoniae* strains (Supplementary Table 1).

The plasmid skeleton is usually composed of a replication regulatory region, distribution system, binding transfer system, and plasmid maintenance region (Novick, 1987; Couturier et al., 1988). Of the 28 multiple-replicon plasmids, five were selected for BLAST analysis, and the five plasmids with high homology (homology ≥ 93%) and the multiple-replicon plasmids with a high abundance of resistance genes were screened for the further analysis of plasmid recombination (Table 1). The plasmid skeleton genes rep, sop/par, tra/trb, and the antibiotic resistance and mobile elements genes were used for comparative structural analysis. For the multiple-replicon plasmid pM1026-3Ar.1 [belonging to IncFIB/IncFII/(IncQ1)], the upstream and downstream gene structure of the *repB* gene was *IS609 insQ-sopB-sopA-repB-intI-parD-ybdN-parB-IS110 tnp*, but the upstream and downstream gene structure of the *repA* gene of the IncFII replicon was *tn5393 tnpA-repA-repA2-pld-IS1 insB*. The incomplete *repA* gene of the IncQ1 replicon occurs within a complex structure composed of 18 resistance genes and 5 mobile elements, and the upstream and downstream gene structure is *repC-repA-IS431mec tnp-Tn21 tnpM-intI1*.

**TABLE 1:** Genome information for representative multiple-replicon plasmids.

**Name**	**Size (kb)**	**G + C content (%)**	**Plasmid type**	**Number of antimicrobial resistance genes**	**Antimicrobial resistance genes**
pBS361-1	104.834	52.83%	IncFII(K)/IncR	2	*mexT, tetG*
pM297-1.2	225.763	52.59%	IncFII(K)/IncQ1	17	*sul2, aph(3*′*)-Ia, aac(3)-IIa, floR, tetG, mphA, mrx, sul1, qnrB2, sul1, emrE, aadA16,arr-3, aac(6*′*)-Ib-cr, bla*_TEM–__1_, *bla*_CTX–M–__3_, *qnrS1*
pM1026-3Ar.1	262.519	51.84%	IncFIB(K)/IncFII(K)/IncQ1	27	*mtrA, oprM, adeB, arlR, nmcR, tetG, catII, mphA, mrx, sul1, emrE, arr-3, catB3, bla*_OXA–__1_, *aac(6*′*)-Ib-cr, aac(3)-IIa, aph(3*′*)-Ia, aph(6)-Id, aph(3″)-Ib, sul2, dfrA12, aadA2, cmlA1, aadA, qacH, sul3, mefB*
pS161-2.2	178.411	50.80%	IncHI1B(pNDM-MAR)/repB	16	*aph(3*′*)-Ia, mphA, mrx, sul1, nmcR, bla_*DHA–*__1_, qnrB4, emrE, arr-3, catB3, bla*_OXA–__1_, *aac(6*′*)-Ib-cr, mtrA, oprM, mexD, arlR*
pS174-1.3	159.310	50.83%	IncFIB(K)(pCAV1099-114)/IncHI1B(pNDM-MAR)/IncQ1	8	*aph(6)-Id, aph(3″)-Ib, sul2, bla*_TEM–__1_, *arlR, adeB, oprM, mtrA*

The upstream and downstream structure of the *repB* gene of the pS174-1.3 plasmid [IncFIB/IncHI1B/(IncQ1)] replicon is *intM*-*int*-*repB*-*sopA*-*sopB*, the upstream and downstream structure of *repA* gene of IncHI1B replicon is IS431mec *tnp*-*other*-*repA*-*yadA*-*other*-Tn903 *tnp*, and the upstream and downstream structure of *repA* gene of IncQ1 replicon is *repC-repA-IS431mec*. The tnp-Tn3 tnpR is also surrounded by a complex structure composed of four resistance genes and two transposase genes. The upstream and downstream gene structure of the *repA1* gene of the pBS361-1 IncFII plasmid (belonging to IncFII/IncR type) replicon is IS431mec *tnp*-*yedK*-*repA1*-*pld*-*aer*-Tn4653 *tnpR*-Tn1721 *tnpA*. The upstream and downstream gene structure of the *repB* gene of the tnpA and IncR replicons is *parM*-*parA*-*other*-*repB*-*other*-*resD*, and the four IncFII/IncR plasmids show high similarity (≥99%), suggesting that they may be different copies of the same plasmid, carrying two resistance genes, *mexT* and *tetG* (Supplementary Table 1).

**FIGURE 4:**
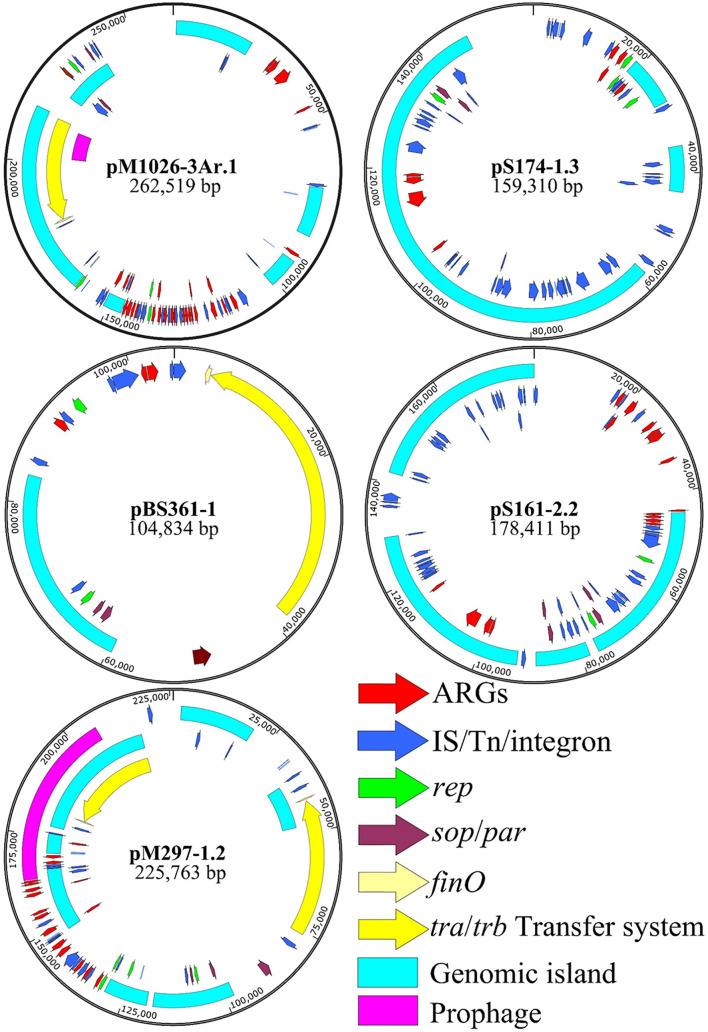
Multiple-replicon plasmid map. pM1026-3Ar.1, pS174-1.3, pBS361-1, pS161-2.2, and pM297-1.2 were selected as representatives, and different colors represent different functional regions.

The upstream and downstream gene structure of the *repA* gene of the pS161-2.2 IncHI1B plasmid (belonging to the IncHI1B/repB type) replicon is Tn1721 *tnpA*-*other*-*other*-*repA*-*other*-*other*-Tn903 *tnp*, and the upstream and downstream gene structure of the *repA* gene of the repB replicon is *sopB*-*sopA*-*repA*-*int*-*intM*. This type of plasmid also has a strong ability to carry resistance genes and has two distribution systems, *sopA-sopB/parA-parB*. The physical distance between the *repA* gene of pM297-1.2 IncFII plasmid and the *repA* gene of IncQ1 is very short (4,038 bp), represented by the structure Tn903 *tnp*-*other*-*repA*(IncFII)-*pld*-*aer*-IS431mec *tnp*-*other*-*repA*(IncQ1)-*repC*. Notably, although the *repA* gene of IncQ1 is not included in the complex structure of the resistance gene and mobile element, it is always followed by the sulfonamide resistance gene sul2 (Figure 4).

The pM279-1.2, pM1026-3Ar.1, and pS174-1.3 plasmids all carried the IncQ1 replicon, and the numbers of resistance genes were 17, 27, and 8, respectively. The ability of these plasmids to carry genes encoding for resistance to β-lactams, aminoglycosides, and sulfonamides was stronger (Table 1), suggesting that the resistance genes-carrying ability may be related to the IncQ1 plasmid. In addition, the pM1026-3Ar.1, pBS361-1, and pM297-1.2 plasmids all exhibited the structure traABCDEFGHIGKLMNOPQRSTUVWXY/trbABCDEFGHIG KLMN-finO, and pM297-1.2 even contained two complete conjugate transfer systems. Mobile elements (insert sequences, transposons, or integrase genes) were found in at least one upstream and downstream of the rep gene in the five plasmids, possibly because mobile elements can act as key fusion sites by mediating the fusion of plasmid fragments to form a complex structure.

**FIGURE 5:**
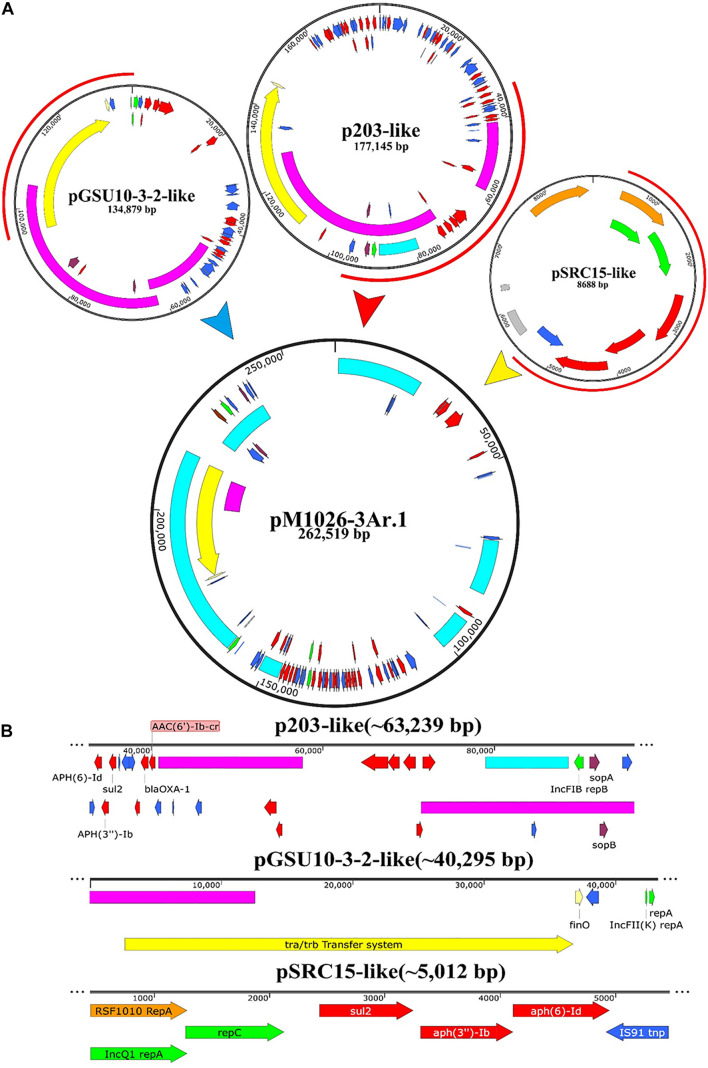
Schematic diagram of the potential fusion process of plasmid pM1026-3Ar.1. **(A)** Fusion process of plasmids P203, pGSU10-3-2, and pSRC15. Red semicircle on the outside of the plasmid indicates the fusion region. **(B)** Specific structure and genetic composition of the fusion fragments provided by plasmids P203, pGSU10-3-2, and pSRC15.

The genomic DNA of the multiple-replicon pM1026-3Ar.1 plasmid contains three replicons IncFIB, IncFII, and IncQ1. An analysis based on BLAST and the PLSDB database showed that three single-replicon plasmids were highly homologous with the pM1026-3Ar.1 replicon (homologous region ≥97%), among which the IncFIB replicon region (32,883–96,121 bp) presented high homology with plasmid p203 of *K. pneumoniae* obtained from human samples in 2018 (GenBank accession number: NZ)_ Cp021166.1, query coverage = 40%); the IncFII replicon region (1,421–94,997 bp) was highly homologous with the pGSU10-3-2 plasmid of *K. pneumoniae* producing the KPC-2 enzyme isolated from a wastewater treatment plant in Japan in 2018 (GenBank accession number: NZ)_ Ap018673.1, query coverage = 31%); and the IncQ1 replicon region (451–5,462 bp) showed high homology with the pSRC15 plasmid of *Salmonella enterica* isolated in 2012 (GenBank accession number: NC)_ 013104.1, query coverage = 1%). These results suggested that the multiple-replicon pM1026-3Ar.1 plasmid may have been formed by the fusion of these three plasmids. According to the structural information of these plasmids, the possible plasmid fusion pattern was drawn (Figure 5).

The full length of plasmid pM1026-3Ar.1 was 262,519 bp. It was speculated that the p203-like plasmid provided ∼63,239-bp gene fragment as the basic plasmid structure, whereas the pGSU10-3-2-like plasmid provided ∼40,295-bp gene fragment of the conjugated transfer region, and the p203- and pGSU10-3-2-like plasmids provided a large number of resistance genes and transportable genes for the pM1026-3Ar.1 mobile element (Figure 5A). The pSRC15-like plasmid provided ∼5,012-bp gene fragment, including ARGs such as *aph(6)-Id*, *aph(3″)-Ib*, *sul2*, *bla*_OXA–__1_, and *aac(6*′*)-Ib-cr*, and a small number of efflux pump genes (Figure 5B). Therefore, under antibiotics pressure, a transformation from multiple single-replicon plasmids to multiple-replicon fusion plasmids is necessary to reduce the cost of survival.

## Discussion

The chromosomal DNA of most *Klebsiella* sp. carries only inherent resistance genes, whereas plasmid-free strains show weak resistance to antibiotics (Figure 1); thus, the inherent resistance genes are not sufficient to endow the strains with a multidrug resistance phenotype, and the resistance genes carried on plasmids are the key to the multidrug resistance phenotype of *Klebsiella* sp. In the present study, we found that the plasmid replicon carried by *Klebsiella* sp. are mainly IncFIB/IncFII/(IncQ1) replicons whether the plasmid is a multiple multiple-replicon plasmid or not (Table 1). A related study showed that *S. enterica* isolated from seven pig samples in Sichuan and Shandong provinces of China contained the strong resistance gene *tetX4* linked to the IncFIB/IncFII type replicon (Bai et al., 2019). The resistance genes *qnrB2*, *aac(6*′*)-Ib-cr*, and *bla*_CTX–M–__3_, related to transposon Tn1548, were co-localized on the IncFII plasmid of *S. enterica* (Du et al., 2012). IncFIB and IncFII plasmids are suitable vectors for *bla*_NDM_, *bla*_KPC_, *bla*_CTX–__M_, *bla*_OXA_, and other extended-spectrum β-lactamases genes (Liu et al., 2019; Musicha et al., 2019; Zhang et al., 2019), and they have the ability to accommodate and stably carry a variety of resistance genes.

In our study, the multiple-replicon plasmid carried by *K. pneumoniae* was shown to be produced mainly *via* the fusion of the single replicons of IncFIB, IncFII, and IncQ1. The bacterial host range of the plasmid is broad, and it can stably exist and be induced. It encodes the resistance genes *sul2*, *strAB*, *tetA*, and *bla*_KPC–__2_, which are associated with sulfamethoxazole, streptomycin, tetracycline, and imipenem, respectively. It was further suggested that the plasmid was responsible for the transmission of resistant strains (Oliva et al., 2017; Martins et al., 2020). pBS361-1, pM297-1.2, pM1026-3Ar.1, pS161-2.2, and pS174-1.3 carried by *K. pneumoniae* are all multiple-replicon plasmids. Thus, it is possible that the single-replicon plasmids provide the framework region of the *rep* gene and *sop*/*par* gene, the conjugate transfer system of the *tra*/*trb*-*finO* gene, and some functional genes and ARGs (Figure 4).

We observed in this study that the ability of multiple replicons to carry resistance genes is significantly stronger than that of single replicon. The number of resistance genes is usually greater than 5, and the number of bases in plasmids increases with the increase in the number of resistant genes. It also further highlights both the danger associated with plasmid-borne resistance and the need to understand why resistance plasmids carry a relatively low cost (Vogwill and MacLean, 2015). In our study, it was found that the multiple-replicon plasmids did not exist in the strains alone, and the majority of *Klebsiella* sp. were found to carry multiple-replicon plasmids, one or more single-replicon plasmids, or unknown plasmids simultaneously. The genome size and the number of genes carried within these plasmids are far smaller than the corresponding values for multiple-replicon plasmids (Supplementary Table 1), and some plasmids even seem to lack genes that are useful to the host (Francino, 2012). In many cases, plasmids impose a fitness cost to their hosts, meaning that the growth rate of plasmid-bearing cells is lower than that of plasmid-free cells. However, this does not fit with the fact that plasmids are ubiquitous in nature nor that plasmids and their hosts adapt to each other very fast—as has been shown in laboratory evolutionary assays (Gama et al., 2018). Even when plasmids are costly, they seem to largely interact in such a way that the cost of two plasmids is lower than the cost of one of them alone (Gama et al., 2018).

In nature, it is very common for bacterial strains to carry multiple-replicon plasmids. The relationship between large plasmids (100–400 kb) and small plasmids (<25 kb) is closer than was expected. Positive epistasis between plasmids minimizes the cost burden of carrying plasmids and improves the stability of plasmids (San et al., 2014). Our study found that the total G + C content of multiple-replicon plasmids did not increase significantly and that the G + C% of a small number of plasmids even decreased (Supplementary Table 1). The G + C content is one of the key factors affecting the adaptive cost of carrying plasmids, and when it decreases, the adaptive cost will also decrease (Yano et al., 2018). Multiple replicons in coordination with maintenance and conjugation regions of various origins would maintain a broad host range and a stable replication at a steady-state plasmid copy number (Qu et al., 2019), which may allow plasmids to avoid incompatibility in a manner similar to the distribution system or provide a way to change the copy number of plasmids to regulate plasmid gene expression, thereby broadening the host range of narrow-host-spectrum plasmids (Pilla and Tang, 2018) and conferring additional resistance. Plasmid or resistance mutations can also improve the adaptability of resistant strains (Silva et al., 2011), which helps to explain why the types of multiple-replicon plasmids found in this study are so complex and still carried a large number of drug-resistance genes and coexisted with different strains.

Among the five plasmids analyzed in this study, the upstream and downstream region of the *rep* genes contained many mobile elements, including insertion sequences, transposons, and integrases (Figure 4); there is a correlation between the formation of multiple replicon plasmids and the presence of mobile elements. Previous studies have shown that the multiple-replicon plasmid formation mechanisms mainly involve insertion sequence (IS)-mediated plasmid fusion and recombination events. The ISPa40 insertion sequence mediated the homologous recombination of plasmids pSa44-CRO and pSa44-CIP, which were integrated into plasmid pSa44-CIP-CRO encoding ciprofloxacin and ceftriaxone-resistant IncI1/IncFIB (Chen et al., 2019). The fusion of the IncFIB type plasmid pBJ114-141 and the IncX3 type plasmid pBJ114-46 may have been mediated by transposition events between the ISKpn19, IS3000, and ISAba125 insertion sequence (Xie et al., 2018). Leelaporn et al. (1996) found that the IS257 insertion sequence element could mediate plasmid integration through an undefined replication transposition mechanism. IS26 is an insertion sequence element that is frequently detected in various resistance-associated plasmids. It can mediate the fusion of IncN1-F33: A-:B-plasmids and phage-like plasmids carrying *mcr-1*. It can also mediate the emergence of the virulent, resistant, and highly transmissible multiple-replicon plasmid pSE380T (IncHI2/IncFIA) of *Salmonella enteritidis* (Wong et al., 2017; He et al., 2019). In this study, plasmid pM1026-3Ar.1 was taken as the representative plasmid to explore the plasmid fusion mode. The p203-like plasmid provided the main frame structure, the pGSU10-3-2-like plasmid provided the conjugated transfer region, the pSRC15-like plasmid provided a small number of resistance genes (Figure 5), and IS1, IS26, IS609, and IS903B were distributed at both ends of these structures, which was consistent with reports of plasmid fusion mediated by insertion sequences (Leelaporn et al., 1996; Wong et al., 2017; Xie et al., 2018; Chen et al., 2019; He et al., 2019). The IS elements carried by the three single plasmids may be the key sites of plasmid fusion, not only mediating the formation of multiple-replicon plasmids but also providing a large number of new gene elements.

We further studied the relationship between multiple-replicon plasmids and bacterial host strains and found that multiple-replicon plasmids showed good compatibility with *K. pneumoniae*. *K. quasipneumoniae* may be a potential host that can carry these plasmids, but *K. variicola* may not be the most suitable host for multiple-replicon plasmids. All three species were found to be widely distributed in *Malayan pangolins* in this study (Figure 3). Multiple-replicon plasmids could be detected in various ST types (Supplementary Table 1), which suggest that there was no significant correlation between multiple-replicon plasmids and the ST type of the strain. According to the global distribution analysis of multiple replicon plasmids pBS361-1, pM297-1.2, pM1026-3Ar.1, pS161-2.2, and pS174-1.3, these plasmids are widely distributed in China and its surrounding countries, and their detection frequency increases every year. Due to the stronger resistance gene-carrying ability and low-cost adaptability of multiple-replicon plasmids, the emergence of a large number of acquired Gram-negative multidrug-resistant strains seems to be closely related to the widespread occurrence of these plasmids, which may be one of the key factors causing severe antibiotic resistance in Asia (Jean and Hsueh, 2011).

## Concluding Remarks

In conclusion, our study confirmed that mobile element-mediated plasmid recombination events could produce multiple-replicon plasmids, thereby promoting the accumulation of various resistance genes among bacteria. These multiple-replicon plasmids have the ability to accommodate multiple ARGs, and the survival ability of the strains carrying these plasmids is improved, but the adaptive cost does not increase significantly. Therefore, there is a close relationship between wild animal-derived isolates and multiple-replicon plasmids, which may be an important survival strategy for coping with external antibiotic pressure. This phenomenon increases the complexity of the antibiotic resistance mechanisms of bacteria, which are responsible for public health hazards that cannot be ignored.

## Data Availability Statement

The sequence information of the sequencing samples used in the study, including the host, collection site and date information, has been submitted to GenBank, and the specific registration number information is shown in Supplementary Table 1.

## Author Contributions

XW and JNZ performed the test, analyzed the data, and drafted and wrote the manuscript. FJ, JY, JQ, ZJ, and HC collected samples and isolated bacteria. GH, GL, XW, JYZ, and CZ assisted in the data analysis. JHQ, BW, and CW edited and read the manuscript. All authors approved the final manuscript.

## Conflict of Interest

The authors declare that the research was conducted in the absence of any commercial or financial relationships that could be construed as a potential conflict of interest.

## Publisher’s Note

All claims expressed in this article are solely those of the authors and do not necessarily represent those of their affiliated organizations, or those of the publisher, the editors and the reviewers. Any product that may be evaluated in this article, or claim that may be made by its manufacturer, is not guaranteed or endorsed by the publisher.

## Footnotes

1
http://rast.nmpdr.org

2
https://cge.cbs.dtu.dk/services/PlasmidFinder/

3
https://isfinder.biotoul.fr/

4
http://bigsdb.web.pasteur.fr/klebsiella/klebsiella.html

5
https://card.mcmaster.ca/
