# Supplementary material for: Multiple-Replicon Resistance Plasmids of Klebsiella Mediate Extensive Dissemination of Antimicrobial Genes
Source: Front Microbiol. 2021 Oct 27;12:754931. doi: 10.3389/fmicb.2021.754931 (PMC8579121; doi:10.3389/fmicb.2021.754931)
Supplement: Supplementary file 1 [file Table_1.docx]

**Supplementary data set 1**

**Table S1. Basic data of the *Klebsiella pneumoniae, Klebsiella quasipneumoniae* and *Klebsiella variicola* strains included in this study**

| Strain name | Collection date | Strain accession | Host | Strain | MLST | Genome size of strain(kb) | Plasmid name | Plasmid accession | Plasmid Finder | Plasmid size of strain(kb) | Plasmid G+C Content (%) | Number of antimicrobial resistance (AMR) genes |
| --- | --- | --- | --- | --- | --- | --- | --- | --- | --- | --- | --- | --- |
| M164-1 | 2019/2/22 | CP063992 | *Manis javanica* | *Klebsiella pneumoniae* | 147 | 5279.777 | N/A | N/A | N/A | N/A | N/A | N/A |
| M169-3 | 2019/2/22 | CP063878 | *Manis javanica* | *Klebsiella pneumoniae* | 101 | 5487.893 | pM169-3.1 | CP063879 | IncFIB(K)/IncFII(pKP91) | 131.081 | 50.89% | 11 |
|  |  |  |  |  |  |  | pM169-3.2 | CP063880 | IncR | 37.458 | 51.21% | 7 |
| S161-2 | 2019/2/22 | CP058544 | *Manis javanica* | *Klebsiella pneumoniae* | 1269 | 5334.536 | pS161-2.1 | CP058545 | IncFII(K) | 104.999 | 52.32% | 3 |
|  |  |  |  |  |  |  | pS161-2.2 | CP058546 | IncHI1B(pNDM-MAR)/repB | 178.411 | 50.80% | 16 |
|  |  |  |  |  |  |  | pS161-2.3 | CP058547 | IncFIB(pKPHS1) | 136.732 | 50.95% | 9 |
| S166-1 | 2019/2/22 | CP063945 | *Manis javanica* | *Klebsiella pneumoniae* | 1910 | 5257.545 | pS166-1.1 | CP063946 | IncFII(K)/IncHI1B(pNDM-MAR)/IncR/IncR/repB | 433.968 | 51.99% | 26 |
|  |  |  |  |  |  |  | pS166-1.2 | CP063947 | novel | 27.799 | 49.82% | 0 |
|  |  |  |  |  |  |  | pS166-1.3 | CP063948 | IncFIA(HI1) | 22.895 | 46.77% | 0 |
|  |  |  |  |  |  |  | pS166-1.4 | CP063949 | IncN/IncN | 83.704 | 51.45% | 4 |
| S174-1 | 2019/2/22 | CP063874 | *Manis javanica* | *Klebsiella quasipneumoniae* | 2354 | 5383.063 | pS174-1.1 | CP063875 | IncX1 | 51.856 | 53.27% | 9 |
|  |  |  |  |  |  |  | pS174-1.2 | CP063876 | IncFIA(HI1)/IncR | 56.886 | 52.95% | 9 |
|  |  |  |  |  |  |  | pS174-1.3 | CP063877 | IncFIB(K)(pCAV1099-114)/IncHI1B(pNDM-MAR)/IncQ1 | 159.310 | 50.83% | 8 |
| S165-1 | 2019/2/22 | CP058548 | *Manis javanica* | *Klebsiella pneumoniae* | 231 | 5355.928 | pS165-1.1 | CP058549 | IncFIB(K)(pCAV1099-114)/IncHI1B(pNDM-MAR) | 169.990 | 50.86% | 11 |
|  |  |  |  |  |  |  | pS165-1.2 | CP058550 | IncFII(K) | 105.000 | 52.33% | 2 |
|  |  |  |  |  |  |  | pS165-1.3 | CP058551 | IncR | 56.644 | 50.66% | 0 |
| M1023-4Ar | 2019/11/18 | CP063851 | *Bos mutus* | *Klebsiella pneumoniae* | 1 | 5141.853 | pM1023-4Ar.1 | CP063852 | IncFIB(K)/IncFII(K)/IncQ1 | 262.508 | 51.84% | 26 |
|  |  |  |  |  |  |  | pM1023-4Ar.2 | CP063853 | IncFIB(pKPHS1) | 110.270 | 49.25% | 1 |
|  |  |  |  |  |  |  | pM1023-4Ar.3 | CP063854 | novel | 77.050 | 52.48% | 1 |
|  |  |  |  |  |  |  | pM1023-4Ar.4 | CP063855 | IncFII(pHN7A8) | 89.958 | 52.76% | 7 |
| N1059-5At | 2019/7/18 | CP063856 | *Bos taurus* | *Klebsiella pneumoniae* | 791 | 5222.128 | pN1059-5At | CP063857 | IncFII(K)/IncQ1 | 92.970 | 52.92% | 18 |
| M1026-3Ar | 2019/7/26 | CP063858 | *Cervus albirostris* | *Klebsiella pneumoniae* | 1 | 5142.109 | pM1026-3Ar.1 | CP063859 | IncFIB(K)/IncFII(K)/IncQ1 | 262.519 | 51.84% | 27 |
|  |  |  |  |  |  |  | pM1026-3Ar.2 | CP063860 | novel | 77.053 | 52.47% | 1 |
|  |  |  |  |  |  |  | pM1026-3Ar.3 | CP063861 | IncFII(pHN7A8) | 89.965 | 52.76% | 4 |
|  |  |  |  |  |  |  | pM1026-3Ar.4 | CP063862 | IncFIB(pKPHS1) | 110.270 | 49.25% | 1 |
| M63-1 | 2018/12/26 | CP063863 | *Ailuropoda melanoleuca* | *Klebsiella pneumoniae* | 628 | 5309.740 | pM63-1 | CP063864 | IncFIB(K)(pCAV1099-114) | 161.463 | 51.52% | 1 |
| M268-3 | 2019/3/14 | CP064319 | *Loxodonta africana* | *Klebsiella variicola* | novel | 5521.406 | pM268-3.1 | CP064320 | novel | 26.136 | 56.41% | 1 |
|  |  |  |  |  |  |  | pM268-3.2 | CP064321 | novel | 28.989 | 58.59% | 0 |
|  |  |  |  |  |  |  | pM268-3.3 | CP064322 | IncFIB(K) | 31.972 | 48.72% | 0 |
| M297-1 | 2019/1 | CP051490 | *Macropus Rfus* | *Klebsiella pneumoniae* | 290 | 5301.757 | pM297-1.1 | CP051491 | IncFIB(K)/IncFII(pKP91) | 222.864 | 51.76% | 9 |
|  |  |  |  |  |  |  | pM297-1.2 | CP051492 | IncFII(K)/IncQ1 | 225.763 | 52.59% | 17 |
| M142-3 | 2018/12 | CP063867 | *Psittacus erithacus* | *Klebsiella variicola* | 3972 | 5615.590 | pM142-3 | CP063868 | IncFIB(K)(pCAV1099-114)/IncFII(K) | 180.701 | 51.89% | 7 |
| M911-1 | 2018/12 | CP064129 | *Aratinga solstitialis* | *Klebsiella pneumoniae* | novel | 5211.192 | pM911-1.1 | CP064130 | novel | 75.711 | 53.74% | 3 |
|  |  |  |  |  |  |  | pM911-1.2 | CP064131 | IncR | 85.824 | 49.18% | 0 |
|  |  |  |  |  |  |  | pM911-1.3 | CP064132 | novel | 21.377 | 47.61% | 0 |
| S141 | 2019/2/28 | CP063871 | *Psittacula alexandri* | *Klebsiella pneumoniae* | 1662 | 5383.698 | pS141.1 | CP063872 | IncFIB(K)(pCAV1099-114)/IncHI1B(pNDM-MAR) | 194.302 | 50.66% | 6 |
|  |  |  |  |  |  |  | pS141.2 | CP063873 | IncFIB(pKPHS1) | 112.160 | 48.72% | 1 |
| S15-2 | 2018/12 | CP064046 | *Eclectus roratus* | *Klebsiella quasipneumoniae* | 2355 | 5330.587 | pS15-2 | CP064047 | IncFIB(K) | 163.717 | 53.10% | 2 |
| S129-1 | 2018/12 | CP063954 | *Sturnus nigricollis* | *Klebsiella variicola* | novel | 5490.156 | N/A | N/A | N/A | N/A | N/A | N/A |
| S130-1 | 2019/3/5 | CP063865 | *Sturnus nigricollis* | *Klebsiella pneumoniae* | 3753 | 5249.027 | pS130-1 | CP063866 | IncFIB(K) | 150.355 | 52.35% | 5 |
| S131-2 | 2019/2/26 | CP063953 | *Gracula religiosa* | *Klebsiella variicola* | novel | 5490.142 | N/A | N/A | N/A | N/A | N/A | N/A |
| S90-2 | 2018/11/27 | CP063881 | *Alectoris chukar* | *Klebsiella pneumoniae* | 629 | 5374.786 | pS90-2.1 | CP063882 | IncFIB(pKPHS1) | 110.388 | 49.18% | 0 |
|  |  |  |  |  |  |  | pS90-2.2 | CP063883 | IncFIA(HI1)/IncFII(K) | 109.675 | 51.09% | 2 |
|  |  |  |  |  |  |  | pS90-2.3 | CP063884 | IncR | 57.825 | 56.48% | 11 |
| M72-2-2 | 2018/11/28 | CP063869 | *Panthera tigris Amoyensis* | *Klebsiella quasipneumoniae* | 3864 | 5442.690 | pM72-2-2 | CP063870 | IncFIB(pKPHS1) | 108.143 | 49.46% | 1 |
| BS329-2 | 2019/2/22 | CP063943 | *Homo sapiens* | *Klebsiella pneumoniae* | 1565 | 5244.603 | pBS329-2 | CP063944 | IncFII(K)/IncR | 104.835 | 52.83% | 2 |
| BS418 | 2019/2/22 | CP063942 | *Homo sapiens* | *Klebsiella quasipneumoniae* | 2144 | 5184.470 | N/A | N/A | N/A | N/A | N/A | N/A |
| BM343 | 2019/2/22 | CP063939 | *Homo sapiens* | *Klebsiella pneumoniae* | 133 | 5358.717 | pBM343.1 | CP063940 | IncFIB(K)/IncFII(K) | 189.015 | 52.68% | 0 |
|  |  |  |  |  |  |  | pBM343.2 | CP063941 | IncR | 68.142 | 50.66% | 0 |
| BS317-1 | 2019/2/22 | CP063936 | *Homo sapiens* | *Klebsiella pneumoniae* | 1035 | 5058.265 | pBS317-1.1 | CP063937 | IncFIB(K)/IncFII(pKP91) | 182.144 | 52.20% | 5 |
|  |  |  |  |  |  |  | pBS317-1.2 | CP063938 | IncR | 62.783 | 53.18% | 7 |
| BS326-3 | 2019/2/22 | CP063934 | *Homo sapiens* | *Klebsiella pneumoniae* | 1565 | 5244.341 | pBS326-3 | CP063935 | IncFII(K)/IncR | 104.836 | 52.83% | 2 |
| BS369-2 | 2019/2/22 | CP063933 | *Homo sapiens* | *Klebsiella variicola* | 4115 | 5518.391 | N/A | N/A | N/A | N/A | N/A | N/A |
| BS375-3 | 2019/2/22 | CP063932 | *Homo sapiens* | *Klebsiella variicola* | 4115 | 5517.087 | N/A | N/A | N/A | N/A | N/A | N/A |
| M186-2 | 2019/2/22 | CP063930 | *Homo sapiens* | *Klebsiella pneumoniae* | 111 | 5237.027 | pM186-2 | CP063931 | IncFIB(K)(pCAV1099-114)/IncHI1B(pNDM-MAR) | 191.041 | 50.21% | 5 |
| S183-1 | 2019/2/22 | CP063927 | *Homo sapiens* | *Klebsiella pneumoniae* | 2158 | 5167.418 | pS183-1.1 | CP063928 | IncFIB(K)(pCAV1099-114) | 208.376 | 49.31% | 8 |
|  |  |  |  |  |  |  | pS183-1.2 | CP063929 | IncFIA/IncFIB(AP001918)/IncFII/IncFII/IncFII(pHN7A8) | 190.929 | 52.40% | 16 |
| S187-1 | 2019/2/22 | CP063926 | *Homo sapiens* | *Klebsiella variicola* | 4394 | 5484.033 | N/A | N/A | N/A | N/A | N/A | N/A |
| S210-3 | 2019/2/22 | CP063925 | *Homo sapiens* | *Klebsiella pneumoniae* | 2668 | 5266.775 | N/A | N/A | N/A | N/A | N/A | N/A |
| BM338-1 | 2019/2/22 | CP063922 | *Homo sapiens* | *Klebsiella pneumoniae* | 35 | 5384.549 | pBM338-1.1 | CP063923 | IncI1 | 82.097 | 50.33% | 0 |
|  |  |  |  |  |  |  | pBM338-1.2 | CP063924 | IncFII(K) | 116.734 | 51.25% | 3 |
| BM404-3-1 | 2019/2/22 | CP064044 | *Homo sapiens* | *Klebsiella quasipneumoniae* | 2355 | 5330.587 | pBM404-3-1 | CP064045 | IncFIB(K) | 163.717 | 53.10% | 2 |
| BM366-1 | 2019/2/22 | CP063921 | *Homo sapiens* | *Klebsiella variicola* | 4115 | 5519.299 | N/A | N/A | N/A | N/A | N/A | N/A |
| BM337-1 | 2019/2/22 | CP063919 | *Homo sapiens* | *Klebsiella pneumoniae* | 1565 | 5292.492 | pBM337-1 | CP063920 | novel | 36.933 | 47.13% | 0 |
| BM374-1 | 2019/2/22 | CP063917 | *Homo sapiens* | *Klebsiella variicola* | novel | 5540.484 | pBM374-1 | CP063918 | novel | 303.115 | 49.69% | 5 |
| BS327-2-1 | 2019/2/22 | CP063916 | *Homo sapiens* | *Klebsiella variicola* | 4115 | 5518.238 | N/A | N/A | N/A | N/A | N/A | N/A |
| M186-1-2 | 2019/2/22 | CP063915 | *Homo sapiens* | *Klebsiella variicola* | 4394 | 5484.339 | N/A | N/A | N/A | N/A | N/A | N/A |
| BM336-2-1 | 2019/2/22 | CP063913 | *Homo sapiens* | *Klebsiella pneumoniae* | 36 | 5406.489 | pBM336-2-1 | CP063914 | IncFIB(K)/IncFII(K)/IncQ1 | 256.775 | 51.94% | 25 |
| BS359-2-1 | 2019/2/22 | CP063912 | *Homo sapiens* | *Klebsiella variicola* | 919 | 5510.850 | N/A | N/A | N/A | N/A | N/A | N/A |
| M186-1 | 2019/2/22 | CP063911 | *Homo sapiens* | *Klebsiella variicola* | 4394 | 5484.445 | N/A | N/A | N/A | N/A | N/A | N/A |
| BS325-2 | 2019/2/22 | CP063910 | *Homo sapiens* | *Klebsiella variicola* | 4115 | 5519.050 | N/A | N/A | N/A | N/A | N/A | N/A |
| M212-2 | 2019/2/22 | CP063908 | *Homo sapiens* | *Klebsiella pneumoniae* | 23 | 5419.625 | pM212-2 | CP063909 | IncHI1B(pNDM-MAR)/repB | 214.844 | 49.75% | 7 |
| BM327-1 | 2019/2/22 | CP063906 | *Homo sapiens* | *Klebsiella pneumoniae* | 1565 | 5299.916 | pBM327-1 | CP063907 | IncFII(K)/IncR | 104.836 | 52.83% | 2 |
| BM334-2 | 2019/2/22 | CP063904 | *Homo sapiens* | *Klebsiella pneumoniae* | 86 | 5409.887 | pBM334-2 | CP063905 | IncHI1B(pNDM-MAR)/repB | 226.993 | 50.24% | 8 |
| BS419-3 | 2019/2/22 | CP063902 | *Homo sapiens* | *Klebsiella quasipneumoniae* | 2355 | 5330.355 | pBS419-3 | CP063903 | IncFIB(K) | 163.712 | 53.10% | 2 |
| BS324-2 | 2019/2/22 | CP063900 | *Homo sapiens* | *Klebsiella quasipneumoniae* | 2558 | 5136.970 | pBS324-2 | CP063901 | IncFIB(K)(pCAV1099-114)/IncHI1B(pNDM-MAR) | 176.438 | 49.93% | 5 |
| BS326-1 | 2019/2/22 | CP063898 | *Homo sapiens* | *Klebsiella variicola* | 347 | 5473.070 | pBS326-1 | CP063899 | IncFIB(K)(pCAV1099-114) | 139.497 | 51.06% | 1 |
| BS419-1 | 2019/2/22 | CP063896 | *Homo sapiens* | *Klebsiella quasipneumoniae* | 2355 | 5329.905 | pBS419-1 | CP063897 | IncFIB(K) | 163.257 | 53.10% | 3 |
| BM378-2 | 2019/2/22 | CP063893 | *Homo sapiens* | *Klebsiella variicola* | 1505 | 5581.862 | pBM378-2.1 | CP063894 | novel | 67.486 | 44.47% | 0 |
|  |  |  |  |  |  |  | pBM378-2.2 | CP063895 | novel | 33.451 | 45.14% | 0 |
| BS359-3 | 2019/2/22 | CP063892 | *Homo sapiens* | *Klebsiella variicola* | 4115 | 5171.077 | N/A | N/A | N/A | N/A | N/A | N/A |
| BS433-2 | 2019/2/22 | CP063890 | *Homo sapiens* | *Klebsiella pneumoniae* | 23 | 5467.009 | pBS433-2 | CP063891 | IncHI1B(pNDM-MAR)/repB | 227.749 | 50.03% | 8 |
| BS325-3-1 | 2019/2/22 | CP063889 | *Homo sapiens* | *Klebsiella variicola* | 4115 | 5598.790 | N/A | N/A | N/A | N/A | N/A | N/A |
| BM419-3 | 2019/2/22 | CP063887 | *Homo sapiens* | *Klebsiella quasipneumoniae* | 2355 | 5339.888 | pBM419-3 | CP063888 | IncFIB(K) | 163.710 | 53.10% | 2 |
| BS361-1 | 2019/2/22 | CP063885 | *Homo sapiens* | *Klebsiella pneumoniae* | 1565 | 5301.662 | pBS361-1 | CP063886 | IncFII(K)/IncR | 104.834 | 52.83% | 2 |

N/A:Not Applicable (No plasmid)
