# Supplementary material for: Multiple-Replicon Resistance Plasmids of Klebsiella Mediate Extensive Dissemination of Antimicrobial Genes
Source: Front Microbiol. 2021 Oct 27;12:754931. doi: 10.3389/fmicb.2021.754931 (PMC8579121; doi:10.3389/fmicb.2021.754931)
Supplement: Supplementary file 2 [file Table_2.docx]

**Supplementary data set 2**

**Table S2 Resistant phenotype and resistant genotype**

|  | | | | | | | | | | |
| --- | --- | --- | --- | --- | --- | --- | --- | --- | --- | --- |
| Strain name | Host | Strain | Antibiotic resistance | **Important Drug-Resistant Gene*** | | | | | | |
|  |  |  |  | **β-Lactam antibiotics** | **Aminoglycoside antibiotic** | **Phenicol antibiotic** | **Fluoroquinolone antibiotic** | **Diaminopyrimidine antibiotic** | **Sulfonamide antibiotic** | **Tetracycline antibiotic** |
| M164-1 | *Manis javanica* | *Klebsiella pneumoniae* | Ampicillin; Cefuroxim; Cefazolin; Ceftriaxone; Cefepime; Chloramphenicol; Levofloxacin; Trimethoprim/Sulfamethoxazole | *bla*_SHV-11_ | - | - | - | - | - | - |
| M169-3 | *Manis javanica* | *Klebsiella pneumoniae* | Ampicillin; Cefuroxim; Cefazolin; Ceftriaxone; Cefepime; Ampicillin/Sulbactam; Piperacillin/Tazobactam; Chloramphenicol; Levofloxacin; Trimethoprim/Sulfamethoxazole | *bla*_SHV-1_; ***bla*_CTX-M-15_**; ***bla*_OXA-1_** | ***aac(6')-Ib-cr***; ***aph(6)-Id***; ***aph(3'')-Ib***; ***aadA*** | ***catII***; ***cmlA5*** | ***qnrB1*** | - | ***sul2***; ***sul1*** | ***tetG*** |
| S161-2 | *Manis javanica* | *Klebsiella pneumoniae* | Ampicillin; Cefuroxim; Cefazolin; Ceftriaxone; Ampicillin/Sulbactam; Piperacillin/Tazobactam; Chloramphenicol; Levofloxacin; Trimethoprim/Sulfamethoxazole | *bla*_SHV-11_; ***bla*_TEM-1_**; ***bla*_CTX-M-15_**; ***bla*_DHA-1_**; ***bla*_OXA-1;_ *bla*_OXA-10_** | ***aph(3')-Ia***; ***aac(6')-Ib-cr***; ***aadA***; ***aph(6)-Id***; ***aph(3'')-Ib*** | ***catB3***; ***cmlA5***; ***floR*** | ***qnrB4*** | ***dfrA13*** | ***sul1***; ***sul2*** | ***tetG*** |
| S166-1 | *Manis javanica* | *Klebsiella pneumoniae* | Ampicillin; Cefuroxim; Cefazolin; Chloramphenicol; Levofloxacin; Trimethoprim/Sulfamethoxazole | *bla*_SHV-11_; ***bla*_OXA-1_**; ***bla*_DHA-1_**; ***bla*_TEM-191_**; ***bla*_TEM-1_**; ***bla*_CTX-M-15_**; ***bla*_CTX-M-15_** | ***aac(6')-Ib-cr***; ***aph(3')-Ia*** | ***catB3***; ***floR*** | ***qnrB4***; ***qnrS1***; ***qepA*** | - | ***sul1***; ***sul1***; ***sul2*** | ***tetG***; ***tetA*** |
| S174-1 | *Manis javanica* | *Klebsiella quasipneumoniae* | Ampicillin; Cefuroxim; Cefazolin; Ceftriaxone; Cefepime; Ampicillin/Sulbactam; Chloramphenicol; Levofloxacin; Trimethoprim/Sulfamethoxazole | *bla*_OKP-B-6_; ***bla*_TEM-1_**; ***bla*_CTX-M-65_**; ***bla*_OXA-10_**; ***bla*_TEM-1_** | *aph(3'')-Ib*; ***aadA***; ***aph(3')-Iib***; ***aadA16***； ***aac(6')-Ib-cr***; ***aph(6)-Id***； ***aph(3'')-Ib*** | ***cmlA5*** | ***qepA***; ***qnrB6*** | - | ***sul1***; ***sul1***； ***sul2*** | ***tetG***; ***tetA*** |
| S165-1 | *Manis javanica* | *Klebsiella pneumoniae* | Ampicillin; Cefuroxim; Cefazolin; Ceftriaxone; Cefepime; Ampicillin/Sulbactam; Chloramphenicol; Levofloxacin; Trimethoprim/Sulfamethoxazole | *bla*_SHV-1_; ***bla*_TEM-1_**; ***bla*_CTX-M-15_** | ***aadA2***; ***aph(3')-Ia*** | ***floR*** | - | ***dfrA12*** | ***sul2*** | ***tetG*** |
| M1023-4Ar | *Bos mutus* | *Klebsiella pneumoniae* | Ampicillin; Cefuroxim; Cefazolin; Ceftriaxone; Cefepime; Gentamicin; Chloramphenicol; Levofloxacin; Trimethoprim/Sulfamethoxazole | *bla*_SHV-1_; ***bla*_OXA-1_**; ***bla*_CTX-M-55_** | ***aadA***; ***aadA2***; ***aph(3'')-Ib***; ***aph(6)-Id***; ***aph(3')-Ia***; ***aac(3)-IIa***; ***aac(6')-Ib-cr***; ***aph(6)-Id***; ***aph(3'')-Ib*** | ***cmlA1***; ***catB3***; ***catII***; ***floR*** | - | ***dfrA12***; ***dfrA13*** | ***sul3***; ***sul2***; ***sul1***; ***sul2*** | ***tetG*** |
| N1059-5At | *Bos taurus* | *Klebsiella pneumoniae* | Ampicillin; Cefuroxim; Cefazolin; Ceftriaxone; Cefepime; Gentamicin; Chloramphenicol; Trimethoprim/Sulfamethoxazole | *bla*_SHV-1_; ***bla*_CTX-M-3_**; ***bla*_TEM-1_** | ***aadA16***; ***aac(6')-Ib-cr***; ***aac(3)-IIa***; ***aph(3')-Ia***; ***aph(6)-Id***; ***aph(3'')-Ib*** | ***floR*** | ***qnrS1*** | ***dfrA5*** | ***sul1***; ***sul2*** | ***tetG*** |
| M1026-3Ar | *Cervus albirostris* | *Klebsiella pneumoniae* | Ampicillin; Cefuroxim; Cefazolin; Ceftriaxone; Cefepime; Gentamicin; Chloramphenicol; Levofloxacin; Trimethoprim/Sulfamethoxazole | *bla*_SHV-1_; ***bla*_OXA-1_**; ***bla*_CTX-M-55_** | ***aac(6')-Ib-cr***; ***aac(3)-IIa***; ***aph(3')-Ia***; ***aph(6)-Id***; ***aph(3'')-Ib***; ***aadA2***; ***aadA*** | ***catII***; ***catB3***; ***cmlA1***; ***floR*** | - | ***dfrA12***; ***dfrA13*** | ***sul1***; ***sul2***; ***sul3***; ***sul2*** | ***tetG*** |
| M63-1 | *Ailuropoda melanoleuca* | *Klebsiella pneumoniae* | - | *bla*_SHV-1_ | - | ***cmlv*** | - | - | - | - |
| M268-3 | *Loxodonta africana* | *Klebsiella variicola* | - | *bla*_LEN-16_ | - | - | - | - | - | - |
| M297-1 | *Macropus Rfus* | *Klebsiella pneumoniae* | Ampicillin; Cefuroxim; Cefazolin; Ceftriaxone; Chloramphenicol; Trimethoprim/Sulfamethoxazole | *bla*_SHV-1_; *bla*_CTX-M-14_; ***bla*_CTX-M-14_**; ***bla*_TEM-191_**; ***bla*_TEM-1_**; ***bla*_CTX-M-3_** | ***aph(3'')-Ib***; ***aph(6)-Id***; ***aph(3')-Ia***; ***aac(3)-IIa***; ***aadA16***; ***aac(6')-Ib-cr*** | ***floR*** | ***qnrS1***; ***qnrB2***; ***qnrS1*** | - | ***sul2***; ***sul1***; ***sul1*** | ***tetG*** |
| M142-3 | *Psittacus erithacus* | *Klebsiella variicola* | - | *bla*_LEN-9_ | - | - | ***emrA***; ***emrB*** | - | - |  |
| M911-1 | *Aratinga solstitialis* | *Klebsiella pneumoniae* | Ampicillin | *bla*_SHV-1_; ***bla*_TEM-191_** | - | - | ***qnrS1*** | - | - | ***tetG*** |
| S141 | *Psittacula alexandri* | *Klebsiella pneumoniae* | - | *bla*_SHV-1_ | - | ***cmlv*** | - | ***dfrA13*** | - | - |
| S15-2 | *Eclectus roratus* | *Klebsiella quasipneumoniae* | Ampicillin; Cefuroxim; Cefazolin; Gentamicin | *bla*_OKP-B-6_ | - | - | - | - | - | - |
| S129-1 | *Sturnus nigricollis* | *Klebsiella variicola* | Ampicillin; Cefuroxim; Gentamicin; Chloramphenicol | *bla*_LEN-34_ | - | - | - | - | - | - |
| S130-1 | *Sturnus nigricollis* | *Klebsiella pneumoniae* | - | *bla*_SHV-11_ | - | - | - | - | - | ***tetG*** |
| S131-2 | *Gracula religiosa* | *Klebsiella variicola* | - | *bla*_LEN-34_ | - | - | - | - | - | - |
| S90-2 | *Alectoris chukar* | *Klebsiella pneumoniae* | Ampicillin; Cefuroxim; Cefazolin; Ceftriaxone; Cefepime; Gentamicin; Chloramphenicol; Levofloxacin; Trimethoprim/Sulfamethoxazole | *bla*_CTX-M-14_; *bla*_SHV-11_ | *aac(3)-Iid*; ***aadA2***; ***aph(3')-Ia***; ***aph(3'')-Ib*** | ***floR*** | - | ***dfrA12*** | ***sul1***; ***sul2*** | ***tetG*** |
| M72-2-2 | *Panthera tigris Amoyensis* | *Klebsiella quasipneumoniae* | Ampicillin | *bla*_OKP-B-6_ | - | - | - | ***dfrE*** | - | - |
| BS329-2 | *Homo sapiens* | *Klebsiella pneumoniae* | Ampicillin | *bla*_SHV-11_ | - | - | - | - | - | ***tetG*** |
| BS418 | *Homo sapiens* | *Klebsiella quasipneumoniae* | Chloramphenicol; Trimethoprim/Sulfamethoxazole | *bla*_OKP-B-7_ | - | - | - | - | - | - |
| BM343 | *Homo sapiens* | *Klebsiella pneumoniae* | Ampicillin; Cefazolin | *bla*_SHV-75_ | - | - | - | - | - | - |
| BS317-1 | *Homo sapiens* | *Klebsiella pneumoniae* | Ampicillin; Cefuroxim; Cefazolin; Ampicillin/Sulbactam; Chloramphenicol; Trimethoprim/Sulfamethoxazole | ***bla*_DHA-1_** | - | - | ***qnrS1*** | - | ***sul1***; ***sul1*** | ***tetG*** |
| BS326-3 | *Homo sapiens* | *Klebsiella pneumoniae* | Ampicillin | *bla*_SHV-11_ | - | - | - | - | - | ***tetG*** |
| BS369-2 | *Homo sapiens* | *Klebsiella variicola* | Ampicillin | *bla*_LEN-16_ | - | - | - | - | - | - |
| BS375-3 | *Homo sapiens* | *Klebsiella variicola* | Cefuroxim; Cefazolin; Ceftriaxone; Cefepime; Amikacin | *bla*_LEN-16_ | - | - | - | - | - | - |
| M186-2 | *Homo sapiens* | *Klebsiella pneumoniae* | Ampicillin; Cefuroxim; Chloramphenicol | *bla*_SHV-11_ | - | ***cmlv*** | - | - | - | - |
| S183-1 | *Homo sapiens* | *Klebsiella pneumoniae* | Ampicillin; Cefuroxim; Cefazolin; Ceftriaxone; Cefepime; Ampicillin/Sulbactam; Gentamicin; Amikacin; Trimethoprim/Sulfamethoxazole | *bla*_SHV-11_; ***bla*_CTX-M-55_**; ***bla*_TEM-1_** | ***aac(6')-Ib-cr***; ***aac(3)-IIa***; ***aadA5*** | ***catB3*** | ***mdtH*** | - | ***sul1***; ***sul1*** | - |
| S187-1 | *Homo sapiens* | *Klebsiella variicola* | - | *bla*_LEN-2_ | - | - | - | - | - | - |
| S210-3 | *Homo sapiens* | *Klebsiella pneumoniae* | Ampicillin | *bla*_SHV-1_ | - | - | - | - | - | - |
| BM338-1 | *Homo sapiens* | *Klebsiella pneumoniae* | Ampicillin; Cefuroxim; Cefazolin; Ceftriaxone | *bla*_SHV-33_；***bla*_TEM-1_**; ***bla*_CTX-M-3_** | - | - | ***qnrS1*** | - | - | - |
| BM404-3-1 | *Homo sapiens* | *Klebsiella quasipneumoniae* | Ampicillin; Cefazolin; Trimethoprim/Sulfamethoxazole | *bla*_OKP-B-6_ | - | - | - | - | - | - |
| BM366-1 | *Homo sapiens* | *Klebsiella variicola* | Ampicillin; Cefazolin | *bla*_LEN-16_ | - | - | - | - | - | - |
| BM337-1 | *Homo sapiens* | *Klebsiella pneumoniae* | Ampicillin; Cefazolin | *bla*_SHV-11_ | - | - | - | - | - | - |
| BM374-1 | *Homo sapiens* | *Klebsiella variicola* | Ampicillin | *bla*_LEN-8_ | - | ***cmlv*** | - | - | - | - |
| BS327-2-1 | *Homo sapiens* | *Klebsiella variicola* | Ampicillin; Cefazolin; Trimethoprim/Sulfamethoxazole | *bla*_LEN-16_ | - | - | - | - | - | - |
| M186-1-2 | *Homo sapiens* | *Klebsiella variicola* | - | *bla*_LEN-2_ | - | - | - | - | - | - |
| BM336-2-1 | *Homo sapiens* | *Klebsiella pneumoniae* | Ampicillin; Cefazolin; Piperacillin/Tazobactam; Gentamicin; Trimethoprim/Sulfamethoxazole | *bla*_SHV-40_; ***bla*_OXA-1_** | ***aac(6')-Ib-cr***; ***aac(3)-IIa***; ***aph(6)-Id***; ***aph(3'')-Ib***; ***aadA2***; ***aadA*** | ***catB3***; ***cmlA1*** | - | ***dfrA12*** | ***sul1***; ***sul2***; ***sul3*** | ***tetG*** |
| BS359-2-1 | *Homo sapiens* | *Klebsiella variicola* | Ampicillin | *bla*_LEN-32_ | - | - | - | - | - | - |
| M186-1 | *Homo sapiens* | *Klebsiella variicola* | - | *bla*_LEN-2_ | - | - | - | - | - | - |
| BS325-2 | *Homo sapiens* | *Klebsiella variicola* | Ampicillin | *bla*_LEN-16_ | - | - | - | - | - | - |
| M212-2 | *Homo sapiens* | *Klebsiella pneumoniae* | Ampicillin; Cefazolin | *bla*_SHV-11_ | - | - | ***mdtH*** | - | - | - |
| BM327-1 | *Homo sapiens* | *Klebsiella pneumoniae* | Ampicillin | *bla*_SHV-11_ | - | - |  | - | - | ***tetG*** |
| BM334-2 | *Homo sapiens* | *Klebsiella pneumoniae* | Ampicillin | *bla*_SHV-1_ | - | - | ***mdtH*** | - | - | - |
| BS419-3 | *Homo sapiens* | *Klebsiella quasipneumoniae* | Ampicillin; Cefazolin | *bla*_OKP-B-6_ | - | - | - | - | - | - |
| BS324-2 | *Homo sapiens* | *Klebsiella quasipneumoniae* | Ampicillin | *bla*_OKP-B-6_ | - | - | - | - | - | - |
| BS326-1 | *Homo sapiens* | *Klebsiella variicola* | Ampicillin; Cefazolin; Piperacillin/Tazobactam | *bla*_LEN-17_ | - | - | - | - | - | - |
| BS419-1 | *Homo sapiens* | *Klebsiella quasipneumoniae* | Ampicillin; Cefazolin; Piperacillin/Tazobactam | *bla*_OKP-B-6_ | - | - | - | - | - | - |
| BM378-2 | *Homo sapiens* | *Klebsiella variicola* | Ampicillin; Cefazolin; Piperacillin/Tazobactam | *bla*_LEN-17_ | - | - | - | - | - | - |
| BS359-3 | *Homo sapiens* | *Klebsiella variicola* | Ampicillin | *bla*_LEN-16_ | - | - | - | - | - | - |
| BS433-2 | *Homo sapiens* | *Klebsiella pneumoniae* | Ampicillin | *bla*_SHV-11_ | - | - | ***mdtH*** | - | - | - |
| BS325-3-1 | *Homo sapiens* | *Klebsiella variicola* | Ampicillin | *bla*_LEN-16_ | - | - | - | - | - | - |
| BM419-3 | *Homo sapiens* | *Klebsiella quasipneumoniae* | Ampicillin | *bla*_OKP-B-6_ | - | - | - | - | - | - |
| BS361-1 | *Homo sapiens* | *Klebsiella pneumoniae* | Ampicillin; Cefazolin; Piperacillin/Tazobactam | *bla*_SHV-11_ | - | - | - | - | - | ***tetG*** |
| *The black font indicates the drug-resistance gene carried by the plasmid | | | | | | | | | | |
